# Mastering your fellowship: Part 1, 2022

**DOI:** 10.4102/safp.v64i1.5481

**Published:** 2022-02-28

**Authors:** Klaus B. von Pressentin, Mergan Naidoo, Gert Marincowitz, Tasleem Ras

**Affiliations:** 1 Division of Family Medicine, School of Public Health and Family Medicine, Faculty of Health Sciences, University of Cape Town, Cape Town, South Africa; 2 Department of Family Medicine, University of KwaZulu-Natal, Durban, South Africa; 3 Department of Family Medicine and Primary Health Care, University of Limpopo, Polokwane, South Africa

**Keywords:** family physicians, FCFP (SA) examination, family medicine registrars, postgraduate training, national exit examination, infectious diseases

## Abstract

The series, ‘Mastering your Fellowship’, provides examples of the different question formats encountered in the written and clinical examinations, that is, Part A of the Fellowship of the College of Family Physicians South Africa (FCFP SA) examination. The series is aimed at helping family medicine registrars (and their supervisors) prepare for this examination.

This section in the *South African Family Practice Journal* is aimed at helping registrars prepare for the Fellowship of the College of Family Physicians South Africa (FCFP SA) Final Part A examination and will provide examples of the question formats encountered in the written examination: multiple-choice question (MCQ) in the form of single best answer (SBA – Type A) and/or extended matching question (EMQ – Type R); short answer question (SAQ), questions based on the critical reading of a journal (evidence-based medicine) and an example of an objectively structured clinical examination (OSCE) question. Each of these question types is presented based on the College of Family Physicians blueprint and the key learning outcomes of the FCFP (SA) programme. The MCQs are based on the 10 clinical domains of family medicine, the SAQs are aligned with the five national unit standards and the critical reading section will include evidence-based medicine and primary care research methods.

This edition is based on unit standard one (critically reviewing new evidence and applying the evidence in practice, principles of self-care and leading a clinical governance team) and unit standard two (evaluate and manage a patient according to the bio-psycho-social approach). The domain covered in this edition is *infectious diseases*. We suggest that you attempt answering the questions (by yourself or with peers and/or supervisors) before finding the model answers online: http://www.safpj.co.za/.

Please visit the Colleges of Medicine website for guidelines on the Fellowship examination: https://www.cmsa.co.za/view_exam.aspx?QualificationID=9.

We are keen to hear about how this series is assisting registrars and their supervisors in preparing for the FCFP (SA) examination. Please email us (editor@safpj.co.za) your feedback and suggestions.

## Multiple-choice question: Single best answer

A 44-year-old patient, on a second-line antiretroviral treatment (ART) regimen for 2 years with a history of treated multi-drug resistant tuberculosis (TB) (2 years ago) and Hepatitis B immune from previous infection, presents with the following blood results (Table 1 and Table 2). She is on Tenofovir (TDF), Emtricitabine (FTC) and Atazanavir/ritonavir (ATV/r):

- *Two months ago*: human immunodeficiency viruses (HIV) viral load 21 600 copies/mL; Log conversion 4.33 Log (copies/mL)
- *Now*: HIV viral load 4713 copies/mL; Log conversion 3.67 Log (copies/mL)

**TABLE 1:** Resistance patterns for the patient (multiple-choice question scenario).

Protease inhibitors (PI)	Major resistance mutations I54V V82A Minor resistance mutations L24I, L10V, K20R, T74S	NRTI resistance mutations	D67N K219E K70R M184V
Atazanavir/r (ATV/r)	Intermediate resistance	Abacavir (ABC)	High-level resistance
Darunavir/r (DRV/r)	Susceptible	Zidovudine (AZT)	Intermediate resistance
Fosamprenavir/r (FPV/r)	Intermediate resistance	Stavudine (D4T)	Intermediate resistance
Indinavir/r (IDV/r)	High-level resistance	Didanosine (DDI)	Intermediate resistance
Lopinavir/r (LPV/r)	High-level resistance	Emtricitabine (FTC)	High-level resistance
Nelfinavir/r (NFV)	High-level resistance	Lamivudine (3TC)	High-level resistance
	-	Tenofovir (TDF)	Low-level resistance
Saquinavir/r (SQV/r)	Intermediate resistance	-	-
Tipranavir/r (TPV/r)	Low-level resistance	-	-
*Integrase inhibitors*	Not tested	*NNRTI resistance mutations*	K103N
Dolutegravir (DTG)	-	Efavirenz (EFV)	High-level resistance
Elvitegravir (EVG)	-	Nevirapine (NVP)	High-level resistance
Raltegravir (RAL)	-	Etravirine (ETR)	Susceptible
	-	Rilpivirine (RPV)	Susceptible

NRTI, nucleoside reverse transcriptase inhibitor.

**TABLE 2:** Stanford score based on the observed resistance profile.

ART drug	ATV	DRV	LPV	ABC	AZT	FTC	3TC	TDF	DTG	ETR
Stanford score	50	0	65	60	55	70	70	15	n/a	0

ART, antiretroviral therapy; ATV, Atazanavir; DRV, Darunavir; LPV, Lopinavir; ABC, Abacavir; AZT, Zidovudine; FTC, Emtricitabine; 3TC, Lamivudine; TDF, Tenofovir; DTG, Dolutegravir; ETR, Etravirine; n/a, not applicable.

Based on these blood results (Tables 1 and 2), what is the most appropriate treatment option for her now?:

- ATV/r; TDF; DTG
- DRV/r; TDF; DTG
- DRV/r; TDF; FTC
- TDF, FTC, DTG
- TDF; FTC; RPV

*Short answer:* c)

### Expanded answer

More persons live with HIV today, and clinicians see more patients failing second-line treatment. Persons living with HIV (PLHIV) on ART have their viral loads (VLs) monitored six months after commencing treatment and then yearly once the VL is suppressed. When patients fail first-line treatment, usually confirmed after enhanced adherence counselling and re-testing of the VL, one usually switches to a second-line regimen without doing resistance testing. The situation becomes much more complicated when patients fail the second-line regimen, especially if this is a PI-based regimen. Treatment failure is confirmed when the VL is greater than 50 copies/mL on two consecutive measurements taken two to three months apart. Detectable VLs less than 1000 copies/mL, followed by an undetectable VL, are termed ‘viral blips’ and do not require an ART regimen change. The reasons for detectable VLs include poor patient adherence, resistance, and inadequate ART drug levels because of altered pharmacokinetics, such as absorption difficulties or drug-drug interactions. The ABCDE approach represents a useful acronym for the busy clinician: adherence, bugs (infections), correct dose, drug interactions, resistance. Transcription errors and recombination result in replicating HIV developing mutations resulting in drug resistance. Prevention of drug resistance requires robust viral suppression by ART. Resistance testing is conducted when the PLHIV develops treatment failure of a second-line regimen allowing the clinician to decide on third-line treatment options. In the public sector, this is usually co-managed with the third-line provincial committee. Protease inhibitors-based regimens are generally more durable and require resistance mutations to develop to result in treatment failure. Treatment failure occurs typically after 2 years of treatment. Therefore, most experts recommend that resistance testing be conducted after the patient has been on a PI-based regimen for at least two years. We currently do genotype drug-resistant testing in the public sector. It is important to do resistance testing while the patient is on a drug regimen for at least four weeks. Wild-type virus tends to predominate when PLHIV stop taking treatment obscuring the resistance test results. Another important concept is that of archived resistance (Nel et al. 2020):

[*A*]fter reverse transcription from its RNA template, HIV inserts a DNA copy into the host genome. Some of the cells that HIV infects are extremely long-lived and essentially provide an ‘archive’ of HIV variants over time. Thus, mutations known to have been present at one point in time can be assumed to be present for the patient’s lifetime, even if they are not visible on the patient’s latest resistance test. (p. 15)

For the patient mentioned above, the resistant patterns are discussed as follow:

**I54V:** a non-polymorphic PI-selected mutation that contributes to reduced susceptibility to each of the PIs except DRV.

**V82A:** a non-polymorphic mutation selected primarily by IDV and LPV. It reduces susceptibility to these PIs and contributes cross-resistance to each of the remaining PIs except DRV and TPV.

**L24I**: a non-polymorphic mutation selected by IDV and LPV. It contributes reduced susceptibility to each PI except DRV and TPV.

**D67N:** a non-polymorphic thymidine analogue mutations (TAM) associated with low-level resistance to Zidovudine (AZT) and Stavudine (D4T). When present with other TAMs, it contributes to reduced susceptibility to ABC, Didanosine (DDI), and TDF.

**K219Q/E:** are accessory TAMS associated with reduced susceptibility to AZT and possibly D4T.

**K70R:** causes intermediate resistance to AZT and possibly low-level resistance to D4T, DDI, ABC and TDF.

**M184V/I:** cause high-level in vitro resistance to Lamivudine (3TC) and FTC and low-level resistance to DDI and ABC. However, M184V/I are not contraindications to continued treatment with 3TC or FTC because they increase susceptibility to AZT, TDF and D4T and are associated with clinically significant reductions in HIV-1 replication.

**K103N:** a non-polymorphic mutation that causes high-level resistance to NVP and EFV.

**FIGURE 1:**
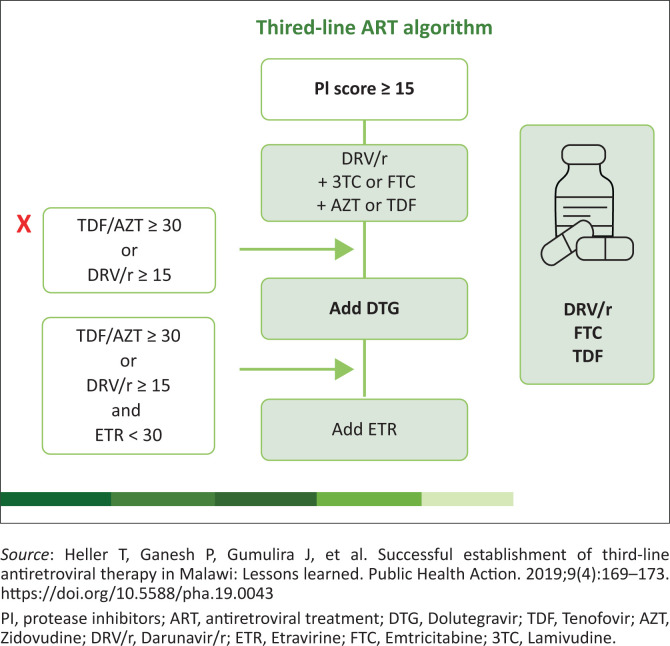
Algorithm for choosing a third-line regimen.

The resistant mutations are also inputted into a Stanford University online database to generate a Stanford total penalty score (PI score in Figure 1). Each drug resistance mutation is assigned a drug penalty score. The total penalty score for each drug from the treatment history is derived by adding the scores for each mutation (and combination of mutations) associated with resistance to that drug. One of five inferred drug resistance or sensitivity levels is then assigned based on the total penalty score.

A penalty score of less than 10 is considered susceptible, 10 to less than 15 is potential low-level resistance, 15–30 is low-level resistance, 30 to less than 60 is intermediate-level resistance, and a score equal to or greater than 60 is high-level resistance. This is a pragmatic approach adopted by clinicians based on available evidence and protects future options for the patient.

Darunavir has the highest barrier to resistance of any PI. For patients with mutations that confer any degree of resistance to DRV (e.g. I50V, L76V and I84V), the dose should be DRV/r 600 mg/100 mg twice daily. For patients without any DRV mutations, the dose is 800 mg/100 mg once daily. Once-daily dosing offers the benefits of reduced pill burden and better side effect profile. Darunavir cannot be co-prescribed with RIF-based TB treatment.

So, if we have to relate the information provided to the case above the following Stanford penalty score can be inferred. In this scenario there is a major PI resistance mutation and the score for LPV/r is > 15 (PI = 65). So, the patient would get DRV/r (PI = O) + 3TC or FTC + AZT (PI = 55) or TDF (PI = 15). In this instance, TDF has a lower penalty score, so the regimen would be TDF/FTC/DRV/r. Because the TDF penalty score is not > 30 and the DRV/r (DRV) penalty score is not > 15, there is no need to add an integrase stand transfer inhibitor (InSTI) Dolutegravir (DTG).

Further reading

- Heller T, Ganesh P, Gumulira J, et al. Successful establishment of third-line antiretroviral therapy in Malawi: Lessons learned. Public Health Action. 2019;9(4):169–173. https://doi.org/10.5588/pha.19.0043
- Nel J, Dlamini S, Meintjes G, et al. Southern African HIV clinicians society guidelines for antiretroviral therapy in adults: 2020 update. S Afr J HIV Med. 2020;21(1):1–39. https://doi.org/10.4102/sajhivmed.v21i1.1115

## Short answer question: The family physician’s role as a care provider and consultant within the domain of infectious diseases

You are working as a family physician in Limpopo. A newly appointed community service doctor (CSD) working at a local clinic, phones you for advice. He asks you about a 45-year-old married man who presented with a headache and fever. At the clinic, he tested positive for malaria. He is also known to be HIV-positive but defaulted treatment. He came to the clinic with his current girlfriend. Your junior colleague asks you for some advice on the management of malaria as he did his internship in Cape Town:

1. What are the important issues that need to be considered to make a clinical assessment? **(8 marks)**
2. What are the important aspects to consider in the management plan? **(3 marks)**
3. What are the important contextual issues that you would like to know about for the future care of this patient at the clinic? **(5 marks)**
4. What ethical dilemmas can potentially be a problem in the ongoing management of this patient? **(4 marks)**

Total: 20 marks

Model answers

**1. What are the important issues that need to be considered to make a clinical assessment? (8 marks)**

- Complicated malaria – as he is HIV-positive and defaulted with an unknown CD4, he should be assessed as complicated malaria. (1 mark)
- Is he local or a traveller? Has he taken prophylaxis? (0.5 marks)
- Other features of complicated malaria (not able to walk, persistent vomiting, jaundice, confused/altered consciousness, respiratory distress, dehydrated, pale, hypoglycaemia/ capillary blood glucose) (4 marks)
- HIV – what is his current status? World Health Organization (WHO) stage? CD4 count/VL? (0.5 marks)
- Previous treatment, adherence history (intermittent or stopped abruptly), reasons for defaulting? (1.5 marks)
- Symptoms of TB? (0.5 marks)

**2. What are the important aspects to consider in the management plan? (3 marks)**

- Needs to be referred to hospital.
- Monitor and stabilise in clinic.
- Start oral dose of artemether-lumefantrine (Co-artem): 4 tablets. These need to be taken with a fatty meal to enhance absorption. Consider the patient’s weight and dose adjustments as required. The first dose should be taken in the clinic and the patient needs to be observed for vomiting. Warn the patient of important side effects (safety netting).

**3. What are the important contextual issues that you would like to know about for the future care of this patient at the clinic? (5 marks)**

- Location and ease of access to the clinic?
- Household members – wife, children, infants who could be HIV exposed. Risk of exposure to HIV or possibly TB and potential adherence support/care?
- Extended family and other social support.
- Sexual partners – girlfriends?
- Employment/income/food security?
- Consider malaria prevention education/measures at the clinic, especially in an endemic area, as there are several non-drug measures which may be considered, for example, light coloured long sleeve clothing at dusk and dawn, use of 50% diethyltoluamide (DEET) products, DEET impregnated sleeping nets, fans, and so on. Important to educate patients who although may be semi-immune in an endemic area, this protective factor is lost as soon as they move out of that area, which may result in more complicated malaria.
- Consider long-term prophylaxis in high-risk groups such as persons living with HIV.

**4. What ethical dilemmas can potentially be a problem in the ongoing management of this patient? (4 marks)**

- Disclosure of HIV status to his wife and girlfriends is the key issue. (1 mark)
- Respect for patient’s autonomy (right to confidentiality) versus justice (rights of third parties to information regarding their health). Beneficence (doing good) and non-maleficence (avoiding harm) should only be discussed in relation to the index patient. Ability to identify the ethical principles. (1 mark)
- If he refuses to discuss his HIV status with either girlfriend or wife, it endangers their lives. They also have the right to the truth and their own health and wellness as well as associated children. The doctor should discuss disclosure with the patient to his girlfriend that is known and present, and if he refuses and it poses no risk to the safety of the girlfriend, tell the patient that you as the doctor is obliged to disclose to the girlfriend (and wife if she is known), especially as they may also be patients known to you or the clinic. Understanding this recommendation. (1 mark)
- The ability of the patient to discuss disclosure should be considered as he is acutely ill and may even be confused. Perceiving this issue. (1 mark)

**Further reading**

- National Department of Health. National guidelines for the treatment of malaria, South Africa [homepage on the Internet]. 2019 [cited 2021 Dec 09]. Available from: https://www.health.gov.za/wp-content/uploads/2020/11/national-guidelines-for-the-treatment-of-malaria-south-africa-2019.pdf
- Brits H. Approach to fever. In: Chapter 5: An approach to common symptoms. In: Mash B, editor. Handbook of family medicine. 4th ed. Cape Town: Oxford University Press, 2017; p. 205–208.
- Adeniyi O, Sogbanmu O, Yogeswaran P. Management of HIV and AIDS. In: Chapter 6: Managing common conditions. In: Mash B, editor. Handbook of family medicine. 4th ed. Cape Town: Oxford University Press, 2017; p. 263–272.
- Moodley K. Chapter 10: Family medicine ethics. In Mash B, editor. Handbook of family medicine. 4th ed. Cape Town: Oxford University Press, 2017; p. 406–429.

## Critical appraisal of quantitative research

Read the accompanying article carefully and then answer the following questions (*total 30 marks*). As far as possible use your own words. Do not copy out chunks from the article. Be guided by the allocation of marks concerning the length of your responses.

- Said RM, Salem GM. Effect of telephone counselling on the knowledge, attitude and practices of contacts of confirmed coronavirus disease 2019 (COVID-19) cases in Egypt. *African Journal of Primary Health Care & Family Medicine*. 2021;13(1):a2852. https://doi.org/10.4102/phcfm.v13i1.2852

1. Critically appraise the strength of the argument for the scientific value of the study – how did they justify doing the study? (6 marks)
2. Critically appraise the authors’ choice of study design, by comparing a randomised vs non-randomised approach to designing a trial to study a public health intervention in terms of benefits and limitations. (6 marks)
3. Critically appraise the piloting of the study instrument in terms of validity and reliability. (6 marks)
4. Critically appraise the sample size and sampling method employed by the researchers. (4 marks)
5. What is the importance of ensuring complete follow-up between the compared groups across the study period? (2 marks)
6. Use a structured approach (e.g. READER) to illustrate what issues arise from this paper when you consider deciding if this study is likely to change your practice. (6 marks)

Model answers

**1. Critically appraise the strength of the argument for the scientific value of the study – how did they justify doing the study? (6 marks)**

- In this paper, the authors argued for the role of contact tracing as an effective public health intervention to control sporadic and clusters of cases before they can spread the infection in pandemic such as COVID-19. The authors mention the presence of ‘numerous studies’ which have shown how health education can play a role in affecting the knowledge, attitudes and practices (KAP theory) of target audiences. The studies cited in the references were conducted in other public health emergencies, such as Ebola and severe acute respiratory syndrome (SARS) outbreaks, in pre-COVID-19 times.
- The authors then make the case for the role of e-health (electronic means of providing health services) and telephone calls as a simple e-health technique, to enhance the reach of health education among people who are experiencing barriers to reach care or receive face-to-face health education.
- The authors argue for telephone calls but admit to the varied effect of this modality and the ambivalence in the literature, when considering its role in telephonic counselling (such as in smoking cessation, a self-management programme for elderly people with osteoarthritis and psychosocial adjustment in women following a cardiac event).
- While there is merit to consider the value of telephonic counselling in health promotion based on the KAP theory in pandemic conditions, it is not clear how the ambivalence cited in the literature supports the argument for telephonic counselling, especially in the examples cited, which represent a range of health promotion scenarios with different levels of complexity and chronicity (from self-management to psychosocial adjustment).
- It may be an oversimplified approach to look at health promotion as an umbrella term without considering the complexities of the condition of interest. Nevertheless, the condition of interest here has social value (COVID-19 pandemic), and the need to find an efficient way to provide health promotion to contacts is justified.
- The scientific argument for the use of telephonic counselling is described to some degree, but a more granular approach to differentiating and comparing health promotion modalities for different conditions would have been better. Understandably, the word count available for a published paper limits the extent of the argument; however, the scope and focus of the engagement with the available literature appears to be limited.

*Additional information (not part of model answer):* The argument for the scientific value of a study is found in the literature review section of a scientific paper which is often integrated in the introduction or background section of the paper. This literature review should address two main issues, namely, what is already known about the topic, and highlighting the gaps in the knowledge field which the research will be addressing. The approaches taken by other researchers should be considered and the information presented with a critical lens (with the goal of evaluating and arguing the value of the contributions cited).

**2. Critically appraise the authors’ choice of study design, by comparing a randomised vs non-randomised approach to designing a trial to study a public health intervention in terms of benefits and limitations. (6 marks)**

- The authors chose to use a non-randomised controlled trial.
- When critically appraising a study design choice, it is important to consider their question and aspects of internal and external scientific validity. While the authors did not clarify the rationale behind their choice of the study design, one may view the study objective of assessing the effect of telephonic counselling on KAP of contacts of COVID-19 confirmed cases as a healthcare intervention.
- Non-randomised studies of the effects of interventions (NRSI) are critical to many areas of healthcare evaluation of public health interventions, but their results may be biased. It is therefore important to understand and appraise their benefits and limitations. Designs of NRSI that can be used to evaluate the effects of interventions include observational studies such as cohort studies and case-control studies in which intervention groups are allocated during usual treatment decisions, and non-randomised studies in which the method of allocation is not randomised. Non-randomised studies can provide evidence additional to that available from randomised control trials about long-term outcomes, rare events, adverse effects, and populations that are typical of real-world practice. For many types of organisational or public health interventions, NRSI are the main source of evidence about the likely impact of the intervention because randomised trials are difficult or impossible to conduct on an area-wide basis. Table 3 compares some of the benefits and limitations of randomised and non-randomised trials.
- For this study, it made sense to select a non-randomised controlled trial design, given the low prevalence of the condition of interest (the study was conducted during the early phase of the pandemic with low community-transmission numbers: March 2020 – April 2020). The researchers wished to study the effect of a telephone-delivered public health intervention consisting of intensive telephone counselling (direct telephone conversations on scheduled alternative days and during any emergency, and continuous WhatsApp contact through text messages or voice calls for any non-urgent questions).

**TABLE 3:** Benefits and limitations between randomised vs non-randomised trials.

Study design	Benefits	Limitations
Randomised trials	The study groups are comparable and balanced with respect to known or unknown risk factors. The intervention is allocated at random or using a quasi-random method of systematic allocation. This allocation counteracts possible biases of the researchers.	Ethical concerns: randomisation is not possible when depriving a participant from a treatment option which may improve his or her condition. Another disadvantage based on ethical reasons is that participants may need to be told that they are part of a trial, which may affect their behaviour and response to questions (this is especially problematic in trials that are not blinded nor use an objective outcome measure). It is not possible to conduct this type of study design when the prevalence of the targeted population is low.
Non-randomised trials	The method of allocation of participants to study groups is not randomised (the assignment to groups is not dependent on chance). Allocation is done by the investigator or the implementer, for example, based on logistics or needs. It may be easier to create study groups with matching characteristics using a non-randomised design.	Study groups may not be comparable. The assignment of participants to study groups may be biased. Baseline characteristics are used to adjust for imbalances. These variables may include demographic and socio-economic characteristics and other covariates potentially associated with outcome and intervention. It is therefore important to measure the outcome of interest at baseline to counteract possible confounders.

*Source:* Colombo D, Cipresso P, Pedroli E, Riva G. Setting-up a clinical trial: Some methodological recommendations. Anuario de Psicología. 2017;47(3):130-139. https://doi.org/10.1016/j.anpsic.2017.12.001; Schmidt WP. Randomised and non-randomised studies to estimate the effect of community-level public health interventions: Definitions and methodological considerations. Emerg Themes Epidemiol. 2017;14(9). https://doi.org/10.1186/s12982-017-0063-5

Note: The model answer is meant to include one advantage and disadvantage for each design.

Total: 6 marks – *The four bullet points (4 marks) and one advantage and disadvantage for each design in Table 3 (2 marks).*

**3. Critically appraise the piloting of the study instrument in terms of validity and reliability. (6 marks)**

- *Validity (3 marks):*
  ■ The authors chose to modify an existing questionnaire (study instrument) used previously among Chinese residents during the ‘rapid rise’ period of the COVID-19 outbreak (see title of reference 12). The instrument is available in Appendix 1 of the paper. The questionnaire was modified by adding new questions in the attitudes and practices sections to ensure enhanced suitability or validity (the accuracy of measuring the outcome of interest: the effect of the telephonic counselling on the KAP of participants in the intervention or experimental group).
  ■ The authors used three Egyptian experts to assist with ensuring content validity (the extent to which the question content of the survey is relevant to the study aim). Relevance was gauged using a four-points rating score.
  ■ However, it is not clear if they ensured face validity (ensuring that the respondents will understand the questions) and construct validity (the extent to which different sections of the survey are closely associated and allow for an in-depth and comprehensive exploration of the topic).
- *Reliability (3 marks):*
  ■ For reliability, the authors piloted the study instrument with the contacts of COVID-19 cases in the first two areas where COVID-19 began to appear. These results were excluded from the main dataset. The authors employed the same methods they planned to use in the main study.
  ■ The statistical test, Cronbach’s alpha, was used to measure internal consistency (reliability), which examines the similarity of answers to different questions about the same concept. This test is especially applicable to Likert-scale or multiple-choice questions. The Cronbach’s alpha result was 0.75, which indicated an acceptable internal consistency. (More information, not necessarily part of the model answer: A general accepted rule is that a Cronbach’s alpha of 0.6–0.7 indicates an acceptable level of reliability, and 0.8 or greater a very good level. However, values higher than 0.95 are not necessarily good, because they might be an indication of redundance).
  ■ The authors did not test inter-rater reliability (to ensure that the questions are delivered in the same way by different researchers), presumably because only the researchers phoned the participants for the telephonic survey (it is not stated explicitly, but one assumes that only the two named authors were conducting the phone calls). It is essential that the outcomes are measured in a reliable way by the study instrument, as unreliability of outcome measurements (here the KAP theory) is one threat that weakens the validity of inferences about the statistical relationship between the ‘cause’ and the ‘effect’ estimated in a study exploring causal effects.

**4. Critically appraise the sample size and sampling method employed by the researchers. (4 marks)**

- *Sample size (2 marks):*
  ■ The authors calculated a sample size of 182 participants by using the improved KAP scores from the small pilot study (16 contacts in the control group and 22 contacts in the experimental group). The authors considered a dropout of 20% to increase the total calculated sample size to 218 participants to be divided equally between the study groups. During the study period (26 March 2020 – 12 April 2020), all COVID-19 contacts appearing in consecutive clusters were assigned to the two study groups (104 participants in each group, 208 in total – see socio-demographic characteristics of the groups in Table 2-A1 of the paper Said & Salem 2021: the first six clusters were the control group, and the next four clusters were the experimental group. The response rate was 100% at baseline.
  ■ This means that the authors were able to recruit more participants than the calculated sample size, which represents a sound result to ensure that the study is adequately powered to answer the research question and detect the difference in measurement between the two groups (the magnitude of the difference between groups is also called the effect size).
- *Sampling method (2 marks):*
  ■ The consecutive approach made sense, especially by assigning the first clusters to the control group to ensure that these participants were not exposed to the public health intervention.
  ■ The baseline socio-demographic characteristics (such as age, gender, rural vs urban residence, education/literacy level and employment status) of the groups were not statistically different. This is important, as the differences between participants included in compared groups constitute a threat to the internal validity of a study exploring causal relationships. If there are differences between participants included in compared groups, there is a risk of selection bias. If there are differences between participants included in the compared groups, it may mean that the ‘effect’ cannot be attributed to the potential ‘cause’, as it may be plausible that the ‘effect’ may be explained by the differences between participants, that is, by selection bias.

**5. What is the importance of ensuring complete follow-up between the compared groups across the study period? (2 marks)**

- In this study, follow-up appeared to be complete with no reported loss to follow-up. Any differences with regard to the loss to follow up between the compared groups may represent a threat to the internal validity of a study exploring causal effects as these differences may provide a plausible alternative explanation for the observed ‘effect’ even in the absence of the ‘cause’ (the treatment or exposure of interest).
- In the situation of loss to follow-up, it would be essential to describe the reasons for loss to follow-up accurately, as well as to analyse the patterns of loss to follow-up, as this may impact on the results.

**6. Use a structured a pproach (e.g. READER) to illustrate what issues arise from this paper when you consider deciding if this study is likely to change your practice. (6 marks)**

The READER format may be used to answer this question:

- Relevance to family medicine and primary care?
- Education – does it challenge existing knowledge or thinking?
- Applicability – are the results applicable to my practice?
- Discrimination – is the study scientifically valid enough?
- Evaluation – given the above, how would I score or evaluate the usefulness of this study to my practice?
- Reaction – what will I do with the study findings?

*The answer may be a subjective response, but should be one that demonstrates a reflection on the change or possible changes within the student’s practice in the South Africa n public healthcare system. It is acceptable for the student to suggest how his or her practice might change, within other scenarios after graduation (e.g. private general practice). The reflection on whether all important outcomes were considered is therefore dependent on the reader’s own perspective (is there other information you would have liked to see?).*

*A model answer could be written from the perspective of the family physician employed in the South African district health system:*

- R: This study is relevant to the African primary care context, as the COVID-19 pandemic has affected all regions of the world and there is a need to look at low-cost public health interventions to influence the KAP of community members, especially regarding the education of COVID-19 contacts on how best to practise infection and control measures.
- E: The authors motivated for the effectiveness of telephonic counselling but acknowledged that the lack of long-term follow-up precluded them from demonstrating maintenance of preventative behaviour over a longer period. The authors recommended that health authorities should be more aware of the potential of telephone counselling during the surveillance of COVID-19 contacts as an accessible, safe and reliable method to improve their KAP. However, this recommendation appeared true during the emerging phase of the pandemic, and it is not known if these recommendations will hold true over time.
- A: In this study, an array of public health interventions has been implemented and health promotion communication has been delivered via a wide range of modalities. It would therefore be difficult to replicate the study in our setting, given the likelihood of confounding covariates. The study setting (Sharkia Governorate, Egypt) is also different from the typical Southern African setting – however, the study setting description is limited with only references to a rural and urban distribution. It would therefore not be possible to generalise the study findings to the wider South African setting.
- D: In terms of discrimination, the study method of a non-randomised controlled trial appears to be appropriate to measure the effects of public health interventions, especially if the condition of interest has a low prevalence, which was the case at the start of the pandemic in the Egyptian study setting (March/April 2020). However, the pandemic has since expanded dramatically with different variants of the virus transmitted at community level and the roll-out of vaccination programmes.
- E: It is unlikely that this study will affect a change in policy direction, largely because of its limitations, but it could help make the case for further research of a more robust design.
- R: The study may be discussed with the local clinical team and used as a basis for reviewing the available options of health promotion interventions. It may also be good to explore the feasibility, acceptability and cost-effectiveness of locally relevant health promotion communication interventions.

**Further reading**

- Mash B, Ogunbanjo GA. African primary care research: Quantitative analysis and presentation of results. Afr J Prim Health Care Fam Med. 2014;6(1):1–5. https://doi.org/10.4102/phcfm.v6i1.646
- Pather M. Evidence-based family medicine. In: Mash B, editor. Handbook of family medicine. 4th ed. Cape Town: Oxford University Press, 2017; p. 430–453.
- Ball L, Barnes K. How to conduct a survey in primary care. In: Goodyear-Smith F, Mash B, editors. How to do primary care research. 1st ed. Boca Raton, FL: CRC Press, 2019; p. 167–175.
- Joannabriggs.org. Critical appraisal tools – JBI [homepage on the Internet]. 2021 [cited 2021 Nov 22]. Available from: https://jbi.global/critical-appraisal-tools
- MacAuley D. READER: An acronym to aid critical reading by general practitioners. Br J Gen Pract. 1994;44(379):83–85.
- Des Jarlais DC, Lyles C, Crepaz N, Trend Group. Improving the reporting quality of nonrandomized evaluations of behavioral and public health interventions: The TREND statement. Am J Publ Health. 2004;94(3):361–366. https://doi.org/10.2105/AJPH.94.3.361
- Sterne JA, Hernán MA, Reeves BC, et al. ROBINS-I: A tool for assessing risk of bias in non-randomised studies of interventions. BMJ. 2016;355:i4919. https://doi.org/10.1136/bmj.i4919
- Schmidt WP. Randomised and non-randomised studies to estimate the effect of community-level public health interventions: Definitions and methodological considerations. Emerg Themes Epidemiol. 2017;14(1):1–1. https://doi.org/10.1186/s12982-017-0063-5

## Objectively structured clinical examination station scenario

### Objective

This station tests the candidate’s ability to care for a patient with long COVID-19 symptoms and persistent tachycardia.

### Type of station

Integrated consultation.

### Role player

Young man or woman.

### Instructions to the candidate

- You are the family physician overseeing the primary care clinic. The following patient comes to see you, having been seen by the medical officer a week ago, and having had some blood tests.
- Please consult with the patient and manage accordingly.
- As this is a chronic mental health consultation, a physical examination is not required.

## Instructions to the examiner

### Objectives

**FIGURE 2:**
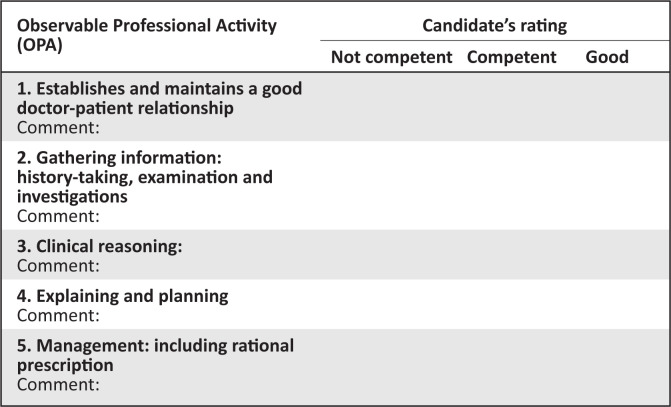
Marking sheet for consultation station.

This station tests the candidate’s ability to care for a patient with long COVID-19 symptoms:

- This is an integrated consultation station in which the candidate has 15 min.
- Familiarise yourself with the assessor guidelines which details the required responses expected from the candidate.
- No marks are allocated. In the marks sheet (Figure 2), tick off one of the three responses for each of the competencies listed. Make sure you are clear on what the criteria are for judging a candidate’s competence in each area.
- Provide the following information to the candidate when requested: see examination findings and investigations below.
- Please switch off your cell phone.
- Please do not prompt the student.
- Please ensure that the station remains tidy and is reset between candidates.

## Guidelines to examiner

### Working definition of competent performance

The candidate effectively completes the task within the allotted time, in a manner that maintains patient safety, even though the execution may not be efficient and well-structured.

### Establishes a good doctor-patient relationship

The *competent candidate* acts within the ethical framework (respects autonomy, justice, non-maleficence, beneficence). In addition, the *good candidate* displays empathy and compassion, acknowledging the patient’s discomfort and the anxiety related to ongoing physical symptoms.

### Gathering information: History-taking, examination and investigations

The *competent candidate* gathers sufficient information to identify current medical issues (*severe functional impairment because of tiredness; fear of COVID-19-related heart condition*) and identify any ongoing biopsychosocial risks. In addition, the *good candidate* explores the patient’s experience, fears (*fear of permanent disability because of persistent drowsiness and lack of motivation; employment prospects*) and expectations, health-seeking behaviour and identifies opportunities for health promotion (*optimising healthy lifestyle choices; explores vaccination attitude*).

### Clinical judgement

The *competent candidate* uses available evidence to make the correct working diagnosis (*long COVID-19, with persistent tachycardia and emotional component)*. The *good candidate* is able to make a comprehensive three-stage assessment (*as for ‘competent’ + fear of disability; impact on occupational function; potential influence of contextual factors*).

### Explaining and planning

The *competent candidate* clearly explains the working diagnosis *[no jargon; comprehensive; simple language]* and possible interventions. In addition, the *good candidate* provides a platform for the patient to engage as an equal partner in sharing information, and decision-making.

### Management

The *competent candidate* uses current evidence-based guidelines to develop a management plan (*symptomatic therapy, avoids over-medicating, information-sharing, provides safety netting and ECG to exclude dysrhythmia; plans for vaccination*). In addition, the *good candidate* develops a comprehensive plan using the biopsychosocial approach (*as for ‘competent’ + counsels the patient on the loss of function and offers assistance with occupational health referral, mentions/refers to the multidisciplinary team; identifies the need for structured follow-up plan*).

### Examination findings and investigations

Vital signs:

- Blood pressure: 125/75; heart rate: 116/min; respiratory rate: 14/min; body mass index: 24; temperature: 36.4 °C
- No jaundice, pallor, lymphadenopathy, clucking, cyanosis, or oedema

Systemic exam:

- Ear nose and throat system: no abnormalities of note.
- Respiratory system: equal air entry bilaterally, normal work of breathing, no abnormalities on auscultation, tidal volume seems normal.
- Cardiovascular system: all pulses present and easily palpable, no bruits over major arteries, heart sounds normal, no murmurs.
- Abdominal system: soft, non-tender.
- Central nervous system: normal gross and fine motor control, sensation intact globally, cognitively normal

Blood results:

- Haemaglobin: 13.4 g/dL
- White cell count: 8.7 (4.0–11.0 × 10E9/L)
- Lymphocytes 1.58 (1.00–4.00 × 10E9/L)
- C-reactive protein: 25
- Creatinine 75
- Thyroid stimulating hormone: 1.2

## Role player instructions

### Appearance and behaviour

A young man and woman

### Opening statement

‘Dr, I was here last week, and your colleague did some blood tests’.

### History

#### Open responses: Freely tell the doctor

- You are 29 years old and recovered from COVID-19 five weeks ago. Had positive nasal swab. Not sure where you got it from. Not vaccinated, but willing to go.
- You have persistent palpitations and feel weak and tired all the time since you had COVID-19.
- You were sick with COVID-19, symptoms lasted 2 weeks, but there was no need for hospitalisation. Your oxygen levels remained more than 95%. The main symptoms were high fever, cough, extreme tiredness, body pains and loss of smell/taste.
- You have been taking Zinc, Vitamin C and Vitamin D every day since you found out you had COVID-19.

#### Closed responses: Only tell the doctor if asked

- Fears:
  ■ You are a trainee manager at a clothing store, and your productivity has taken a massive hit because of your very low energy levels.
  ■ You worry that this is a permanent post-COVID-19 condition and that you may feel like this forever.
  ■ Very worried that COVID-19 damaged your heart.

### Social history

- Single, living alone, and not in a romantic relationship at the moment.
- You used to play soccer with friends twice a week but cannot any longer.
- Friends are supportive on social media but hardly visit – they have their own lives.

#### If the doctor asks specific questions

- Mood:
  ■ Feel worried about this tiredness but want to get back to normal to start enjoying life again. Hopeful that there will be a solution.
- You enjoy soccer – played about 3–4 h per week pre-COVID, now no energy for this.
- Habits: weekends are usually about partying and lots of alcohol, occasionally cocaine. Smokes about 10 cigarettes/day since age 20 years.

#### Further reading

- Mendelson M, Nel J, Blumberg L, et al. Long Covid: An evolving problem with an extensive impact. S Afr Med J. 2021;111(1):10–12. https://doi.org/10.7196%2FSAMJ.2020.v111i11.15433

